# Hackflex library preparation enables low-cost metagenomic profiling

**DOI:** 10.1093/ismeco/ycae075

**Published:** 2024-05-29

**Authors:** Samantha L Goldman, Jon G Sanders, Daniel D Sprockett, Abigail Landers, Weiwei Yan, Andrew H Moeller

**Affiliations:** Department of Ecology and Evolutionary Biology, Cornell University, 215 Tower Rd, Ithaca, NY 14850, United States; Department of Ecology and Evolutionary Biology, Princeton University, 301 Guyot, Princeton, NJ 08540, United States; Department of Ecology and Evolutionary Biology, Cornell University, 215 Tower Rd, Ithaca, NY 14850, United States; Department of Ecology and Evolutionary Biology, Cornell University, 215 Tower Rd, Ithaca, NY 14850, United States; Department of Ecology and Evolutionary Biology, Cornell University, 215 Tower Rd, Ithaca, NY 14850, United States; Department of Ecology and Evolutionary Biology, Cornell University, 215 Tower Rd, Ithaca, NY 14850, United States; Department of Ecology and Evolutionary Biology, Cornell University, 215 Tower Rd, Ithaca, NY 14850, United States; Department of Ecology and Evolutionary Biology, Princeton University, 301 Guyot, Princeton, NJ 08540, United States

**Keywords:** microbiome, microbial ecology, community composition, Opentrons, liquid-handling robots, Zymo

## Abstract

Shotgun metagenomic sequencing provides valuable insights into microbial communities, but the high cost of library preparation with standard kits and protocols is a barrier for many. New methods such as Hackflex use diluted commercially available reagents to greatly reduce library preparation costs. However, these methods have not been systematically validated for metagenomic sequencing. Here, we evaluate Hackflex performance by sequencing metagenomic libraries from known mock communities as well as mouse fecal samples prepared by Hackflex, Illumina DNA Prep, and Illumina TruSeq methods. Hackflex successfully recovered all members of the Zymo mock community, performing best for samples with DNA concentrations <1 ng/μL. Furthermore, Hackflex was able to delineate microbiota of individual inbred mice from the same breeding stock at the same mouse facility, and statistical modeling indicated that mouse ID explained a greater fraction of the variance in metagenomic composition than did library preparation method. These results show that Hackflex is suitable for generating inventories of bacterial communities through metagenomic sequencing.

## Introduction

The ability to sequence the entire complement of DNA present in an environmental sample (i.e. metagenomic shotgun sequencing) has rapidly expanded knowledge of the structure and function of microbial communities. However, metagenomic shotgun sequencing remains cost prohibitive for many studies, prompting development of methods that reduce the cost of metagenomic DNA library preparation [1–3]. One of these methods, Hackflex, adapts the Illumina DNA Prep protocol (formerly labeled Nextera Flex) by diluting bead-linked transposases during tagmentation and using commercially available reagents, yielding libraries at 1/14th the cost [3].

Hackflex has been validated and used in the context of whole-genome sequencing for bacterial isolates [4–10] and amplicon sequencing [11]. Although Hackflex has been used in metagenomic analyses, its accuracy has only been tested in the context of simple mock communities containing seven species of known relative abundances [12, 13]. The utility of Hackflex for metagenomic shotgun sequencing of complex microbial communities from natural sources has not been robustly evaluated.

Here, we tested the effectiveness of Hackflex for metagenomic sequencing by assessing its ability to accurately recover bacterial DNA from a known mock community and to profile bacterial communities in mouse fecal samples. We compare Hackflex to the higher-cost Illumina DNA Prep and TruSeq library preparation methods. We show that Hackflex libraries accurately recover known Zymo mock communities and identify template DNA concentrations that minimize observed biases in relative abundance estimates. When applied to mouse fecal DNA, Hackflex libraries recapitulated results obtained from costlier Illumina DNA Prep and TruSeq libraries and were sufficiently sensitive to differentiate the metagenomes of individual mice from the same inbred line reared in a common environment. These results support the utility of Hackflex for metagenomic studies.

## Results and discussion

### Hackflex recapitulates Zymo mock communities

To assess the accuracy of metagenomes sequenced from libraries prepared by Hackflex (Supplementary Methods), we sequenced Hackflex libraries prepared from the ZymoBIOMICS Microbial Community DNA Standard (Catalog # D6306) —a cocktail of DNA from eight bacterial and two fungal species mixed at known proportions—using a range of template DNA concentrations. In total, 18 Zymo mock DNA communities were sequenced at seven different DNA template concentrations generated by serial 1:2 dilutions of each sample (Table S1; Supplemental Methods). Details about library fragment size and sequencing depth are presented in Supplementary Results.

**Figure 1:**
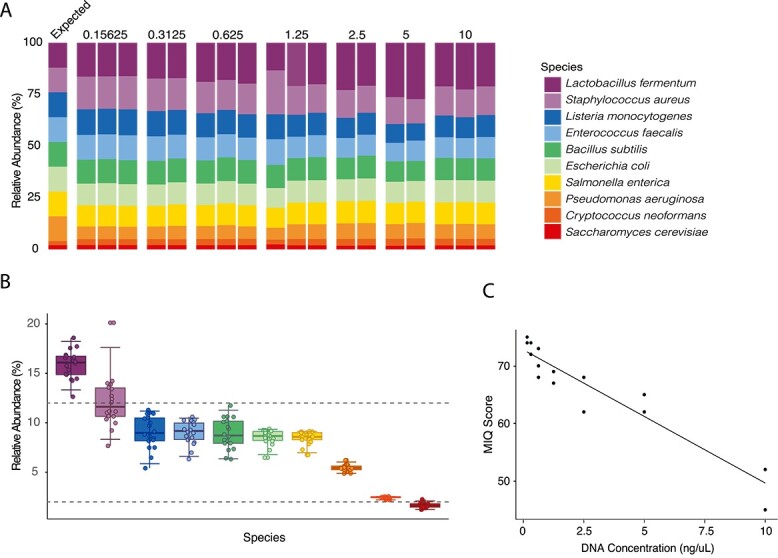
**Hackflex recovers mock community metagenomes.** (A) Normalized taxa barplots show expected (left-most bar) and observed relative abundances for mock-community members. Expected relative abundances were the same for each DNA concentration (ng/μL). Colors denote microbial species as indicated by the key. (B) Boxplots show observed and expected relative abundances for each taxon in each sample. Colors correspond to those in (A). Points represent relative abundances of microbial species. 95% confidence intervals are shown for each species. Horizontal dashed lines show the expected relative abundances for each species of bacteria (top line, 12%) or eukaryote (bottom line, 2%). Points above or below the dashed lines indicate over- or under-representation, respectively, of the species compared to its expected relative abundance. (C) Scatterplot shows MIQ scores and DNA concentrations for each sample. Points represent libraries generated from Zymo mock DNA. Line indicates best-fit regression.

To assess the accuracy of Hackflex, we next used a custom Kraken2 [14] database containing genomes from all Zymo mock community members to measure the difference between the observed and known relative abundance for each community member. Comparing observed to expected relative abundances indicated that Hackflex successfully replicated the expected relative abundances of the bacterial (12%) and fungal (2%) components of the Zymo mock DNA community within one order of magnitude for each sample (Fig. 1A and B). The percentages of reads that mapped to the custom database with minimap2 [15] ranged from 96.85 to 98.34% (mean = 97.93%) (Table S2). Mapping the 2% unclassified reads against the Kraken2 databases indicated that >96% of unclassified reads for each sample mapped to conspecific strains of the species included in the Zymo database. These results suggest that a combination of sequencing errors and minor strain variation can explain the presence of these unmapped reads and indicate minimal contamination in these experiments.

We observed an overrepresentation of *Lactobacillus* and an underrepresentation of *Listeria*, *Bacillus*, *Enterococcus*, *Escherichia*, *Salmonella*, and *Pseudomonas* (Fig. 1B) (95% confidence intervals of measured abundances did not overlap with expected abundances). This divergence between observed and expected relative abundances could be due to errors during library preparation and sequencing, but it could also be driven by errors during the manufacturing of the Zymo mock community. To address the performance of Hackflex, for each Zymo metagenome we calculated Measurement Integrity Quotient (MIQ) scores, which assess bias by calculating deviations from the expected relative abundance of each taxon while allowing for variance inherent in the manufacturing of mock communities [16]. Results from these analyses indicated that Hackflex yielded passing scores (i.e. >60) for 16/18 samples, with only libraries prepared from DNA samples at highest concentration (10 ng/μL) failing (mean = 66.778, median = 68) (Fig. 1C, Table S3, Supplementary Fig. S1). Hackflex MIQ scores were significantly negatively associated with DNA concentration (R^2^ = 0.876; *P* = 1.19e-8) (Fig. 1C, Table S2), but not GC content (Table S3). One explanation for the negative association with DNA concentration is that DNA molecules may compete more for DNA tagmentase when DNA concentrations are high, exacerbating any underlying biases. We additionally calculated the MIQ scores for samples from a previous study which prepared five Illumina DNA Prep libraries from the Zymo mock DNA community [17] (mean MIQ score = 86.8, median = 86) (Table S2). Therefore, at low input DNA concentrations, Hackflex yielded MIQ scores ~86% as high as those obtained from Illumina DNA Prep, while allowing savings of over 10-fold lower reagent costs. These results indicate that error resulting from Hackflex library preparation can be reduced by diluting template DNA to ~0.15 ng/μL.

### Hackflex corroborates Illumina DNA Prep and TruSeq when applied to mouse gut metagenomes

**Figure 2:**
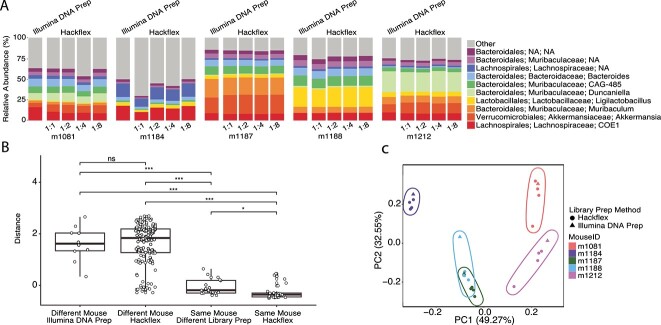
**Hackflex recovers individual signatures in mouse gut metagenomes**. (A) Taxa barplots show relative abundances of microbial taxa observed in mouse metagenomes sequenced from libraries prepared with Illumina DNA Prep or Hackflex. Colors denote microbial genera as indicated by the key. (B) Boxplots show the DEICODE Aitchison dissimilarities between pairs of samples from different mice prepared with Illumina DNA Prep, different mice prepared with Hackflex, the same mice prepared with different library methods, and the same mouse prepared with Hackflex. Points represent pairwise comparisons between samples. Asterisks and “ns” indicate significance of differences between boxplots based on permutation t-tests for non-independent samples; Bonferroni-corrected *P*-value <0.05 *; < 0.001 ***; > 0.05 ns. (C) Principal coordinate analysis plots show Robust Aitchison dissimilarities among samples. Circles represent Hackflex-prepared libraries and triangles represent Illumina DNA Prep-prepared libraries. Colors denote individual mice as indicated by the key.

We also tested the accuracy of Hackflex for biological samples (fecal samples) by comparing its performance with Illumina DNA Prep (Fig. 2). We prepared 25 libraries from five fecal samples from different mice. To prioritize technical replication for Hackflex libraries, we prepared four serial 1:2 dilutions per Hackflex-prepared sample (Fig. S1, Table S1). Details regarding library fragment size and sequencing depth are presented in the Supplementary Results. Illumina DNA Prep samples were used as a reference against which Hackflex samples were compared for accuracy.

Hackflex performed comparably to Illumina DNA Prep in recovering metagenomes from mouse fecal samples (Fig. 2A). PERMANOVA based on Woltka [18] taxonomic profiles generated for all samples indicated that mouse ID was a stronger driver of community variation (R^2^ = 0.222) compared to library prep method (Illumina DNA Prep vs Hackflex) (R^2^ = 0.053) (Table S3). Pairwise robust Aitchison DEICODE [19] comparisons between samples showed that taxonomic profiles of samples collected from the same mouse but whose libraries were prepared by different methods were more similar than those of libraries collected from different mice but whose libraries were prepared by the same method (Fig. 2B) (Bonferroni-corrected *P*-value <0.001; pairwise permutation t-test for non-independent samples). Furthermore, principal coordinate analysis showed that these samples clustered by mouse individual, not library preparation method (Fig. 2C). The two microbiota profiles that were most similar came from cagemates, which were expected to share microbes (Table S4). Qualitatively identical PERMANOVA results were observed in analyses based on Kraken2/Bracken profiles. Taxonomy relative abundances from Woltka and Kraken2 are presented in Tables S5, S6, and S7. Rarefaction with nonpareil [20] did not detect a significant difference in diversity (*N_d_*) as a function of library preparation strategy (mean = 18.23 and 17.67 for Illumina DNA prep and Hackflex, respectively; t-test and Wilcoxon test *P*-values >0.1), and estimates of taxa relative abundances were not biased as a function of microbial domain, gram status, GC content, or genome size (Tables S3 and S8, Fig. S3). In addition, we tested the accuracy of Hackflex by comparing results obtained from five additional mouse fecal samples for which libraries were prepared with Hackflex and Illumina TruSeq, revealing qualitatively identical results (Supplementary Results, Fig. S4).

This study shows that Hackflex represents an effective, low-cost library preparation method for shotgun metagenomics. One limitation of this study is that we did not assess environmental samples (e.g. soil, water), which can contain higher levels of microbial diversity (compared to mouse fecal samples) that may require use of undiluted library preparation strategies. Here, we show that Hackflex was able to recover known mock communities and individual-host signatures in the gut microbiota among mice of the same genotype reared in a common environment, and that biases of the method can be mitigated by dilution of template DNA to concentrations as low as ~0.15 ng/μL. Overall, this study shows that Hackflex can reduce the costs of inventorying microbial taxa and estimating their relative abundances within complex microbial communities.

## Supplementary Material

SupplementaryFig1_ycae075

SupplementaryFig2_ycae075

SupplementaryFig3_ycae075

SupplementaryFig4_ycae075

Supplementary_Tables_ycae075

Supplemental_Methods_ycae075

## Data Availability

Sequence data were uploaded to NCBI archive under accession number PRJNA1114129. The scripts used to generate figures are available at https://github.com/samanthagoldman/hackflex_validation/ and at the Zenodo record https://zenodo.org/doi/10.5281/zenodo.10965899.
