# Supplementary figures and images for: Hackflex library preparation enables low-cost metagenomic profiling
Source: ISME Commun. 2024 May 29;4(1):ycae075. doi: 10.1093/ismeco/ycae075 (PMC11190725; doi:10.1093/ismeco/ycae075)

Dilution (ng/uL)

10

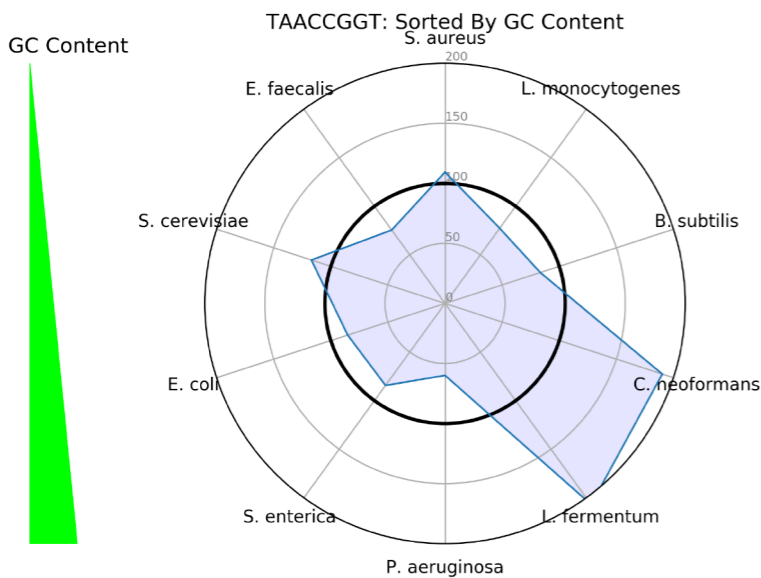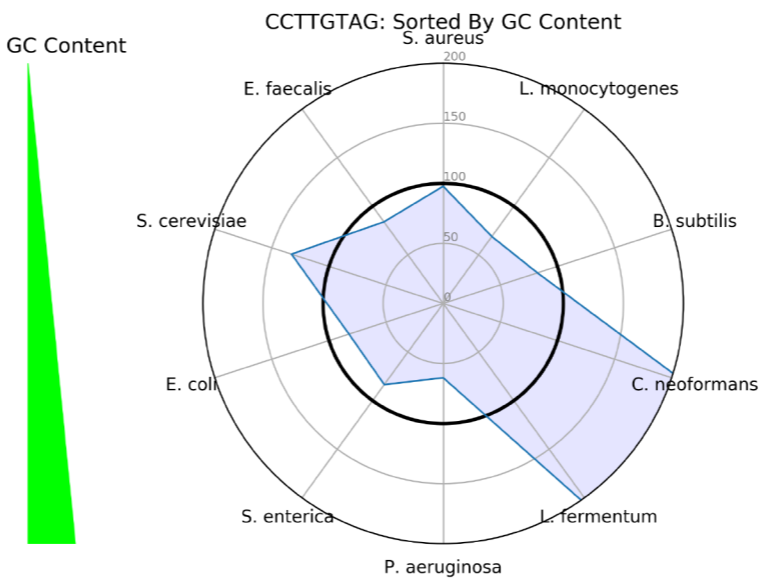

5

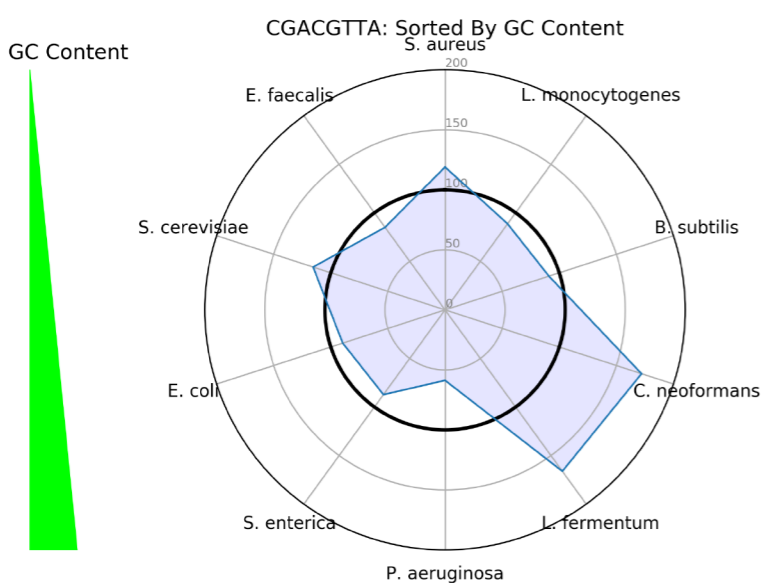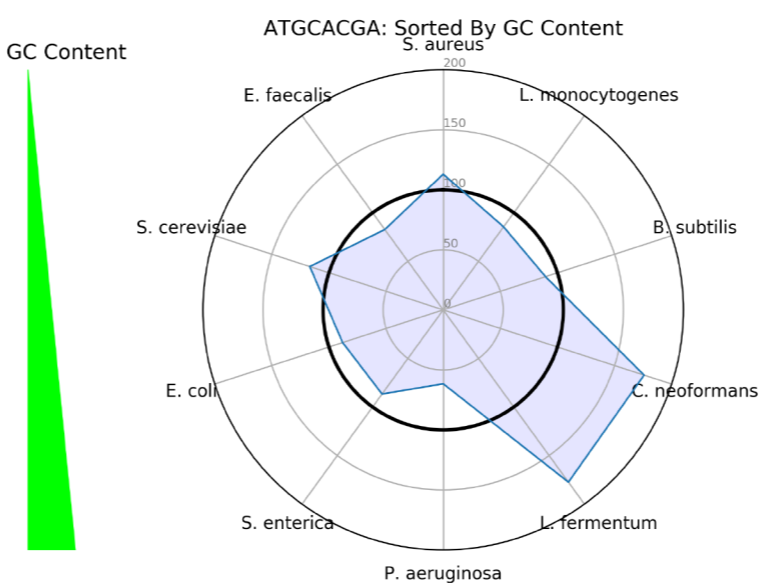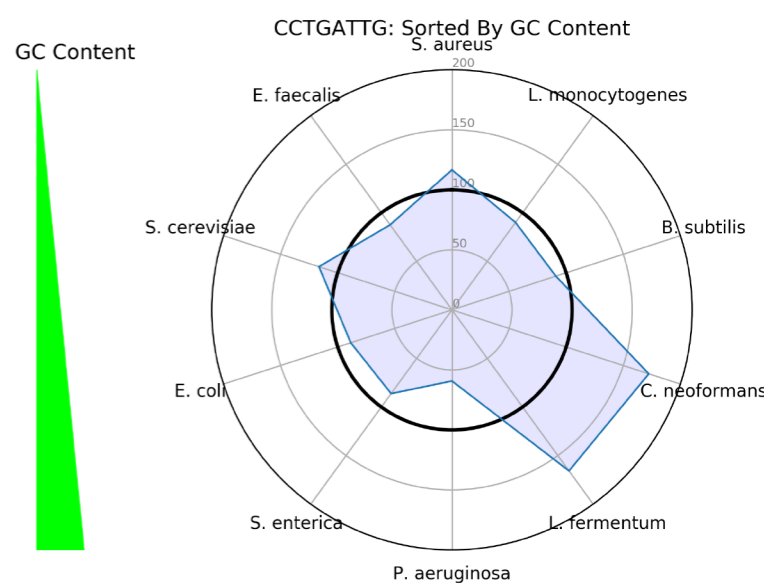

2.5

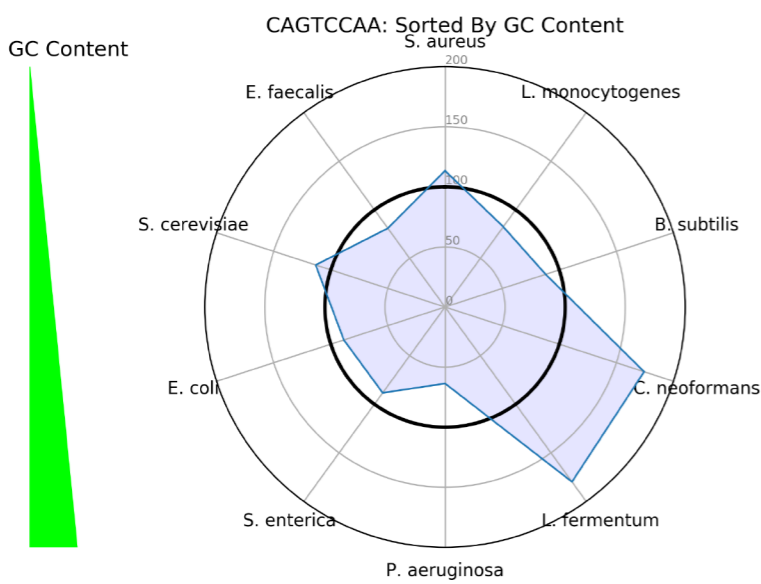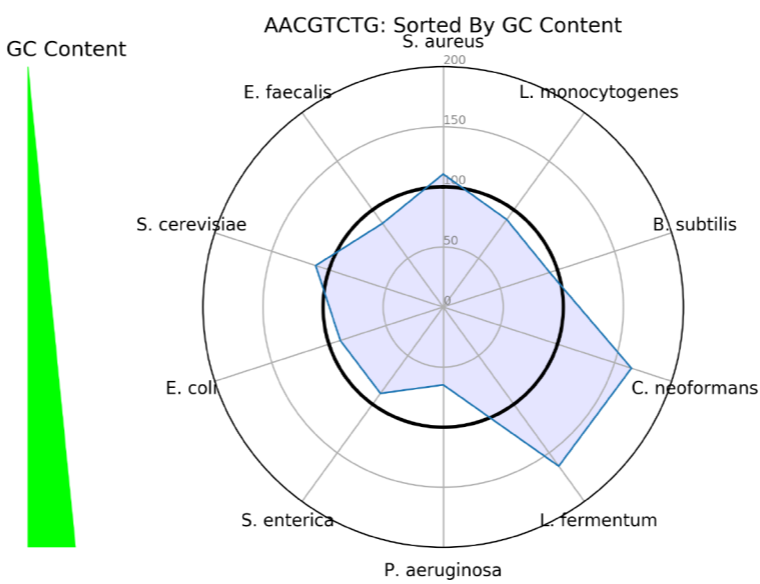

1.25

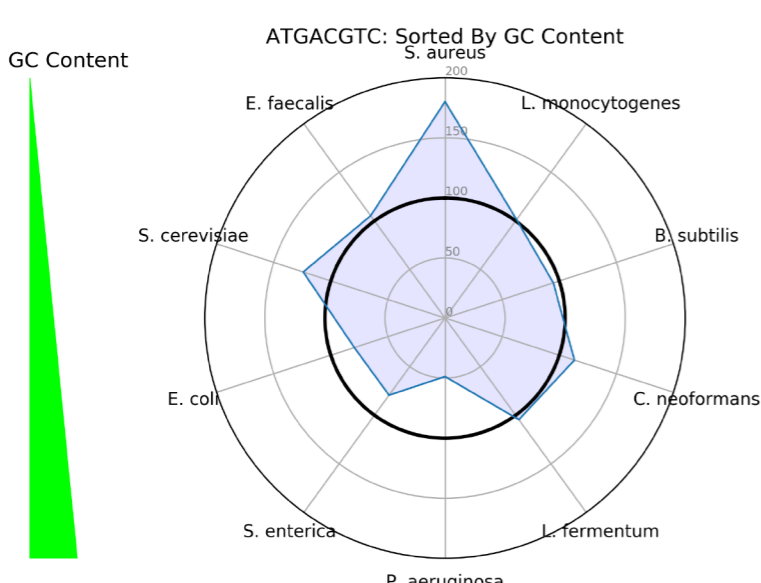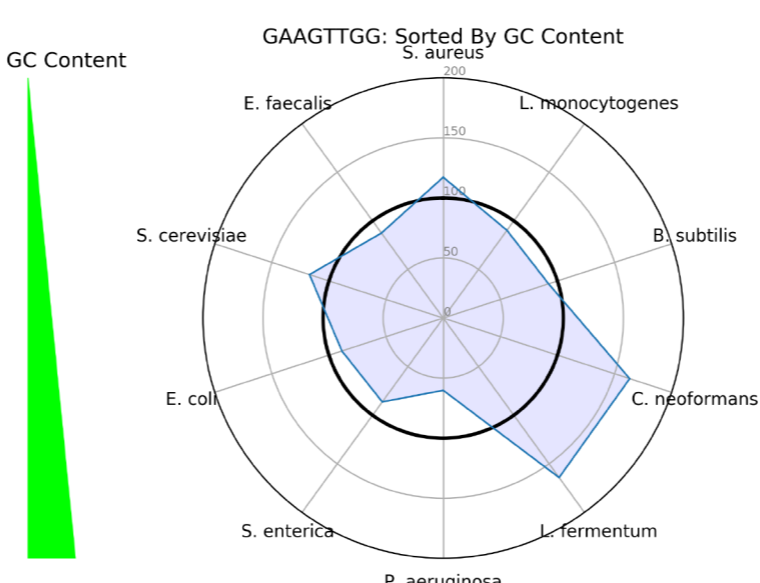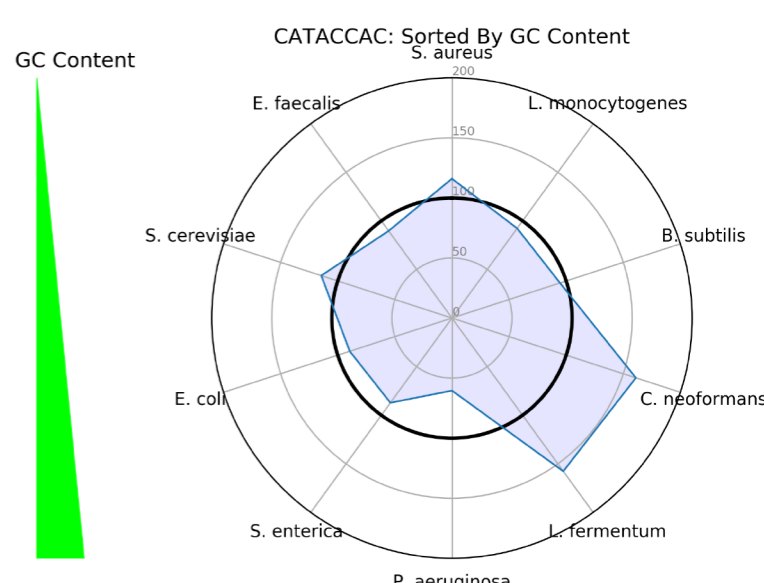

0.6125

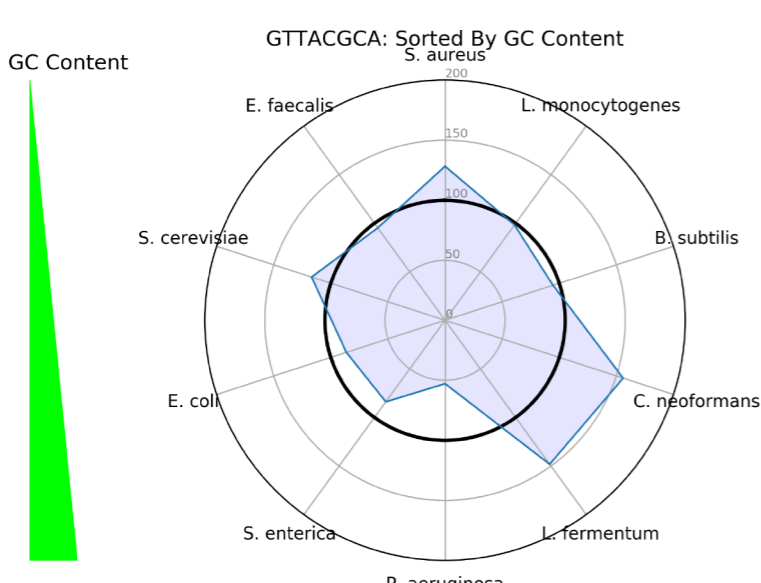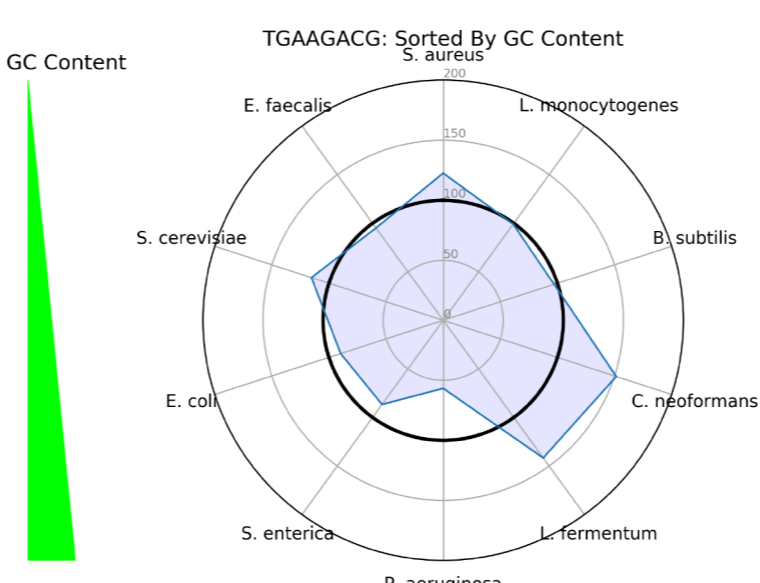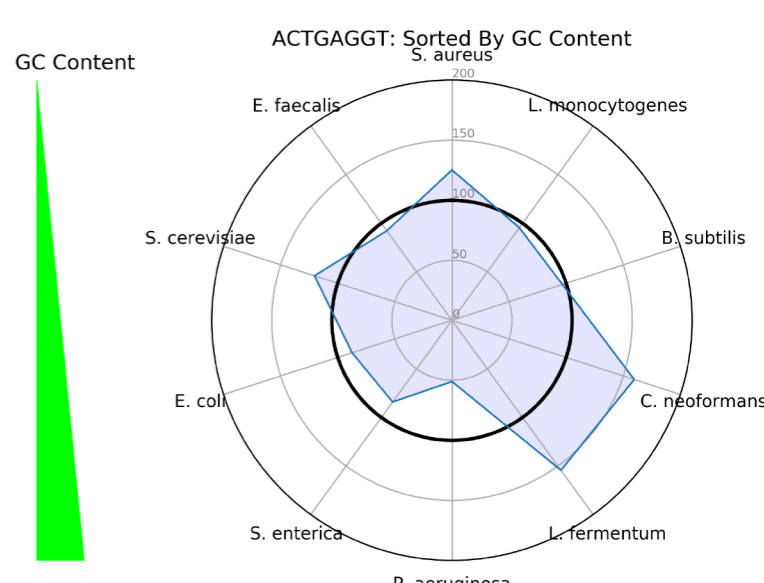

0.3125

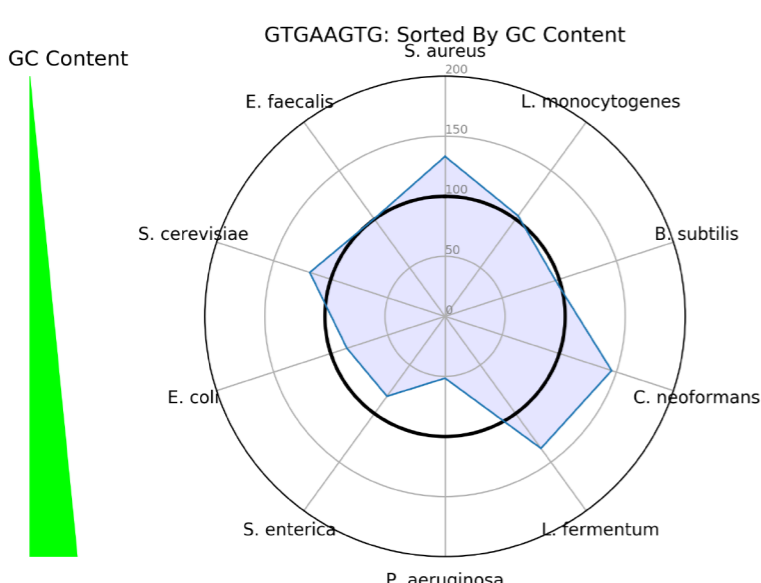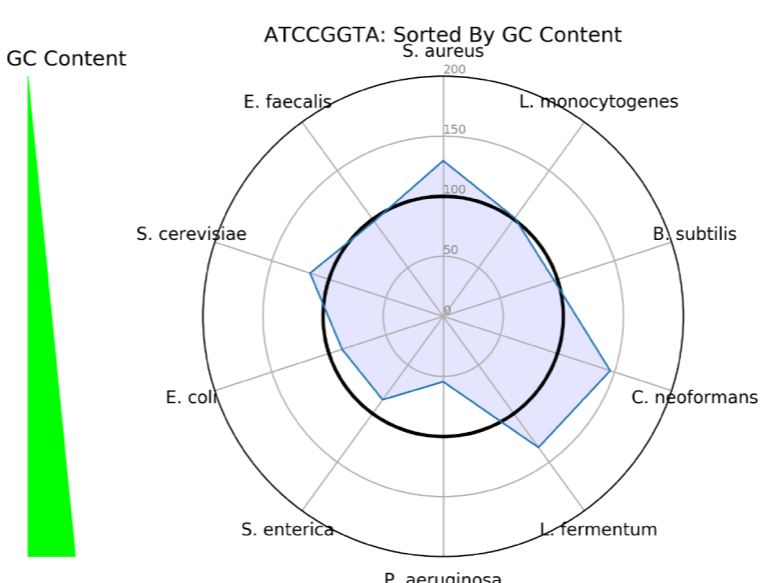

0.15625

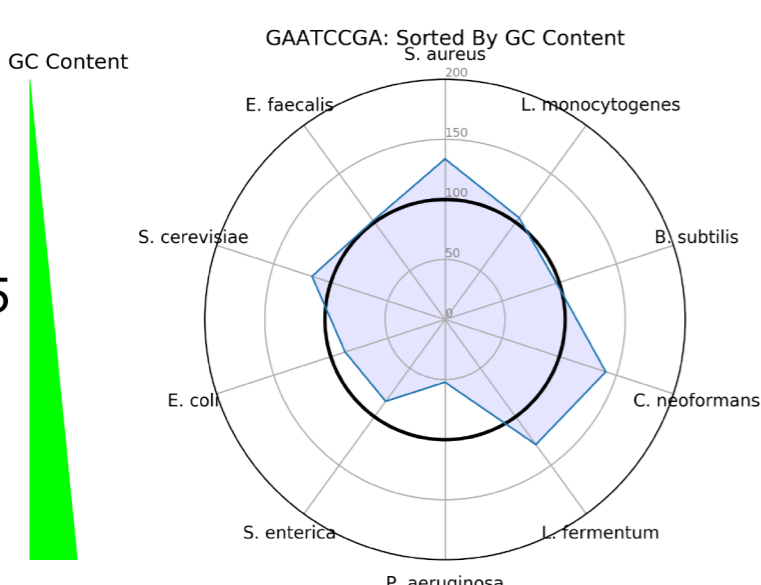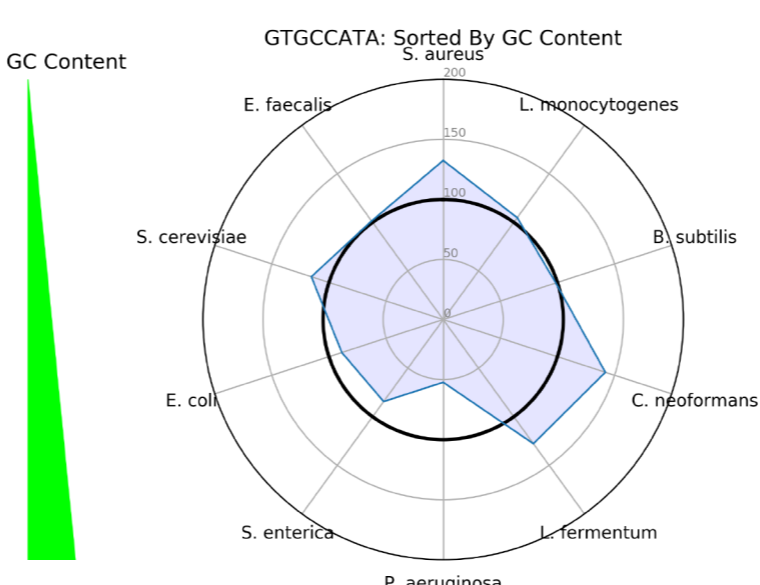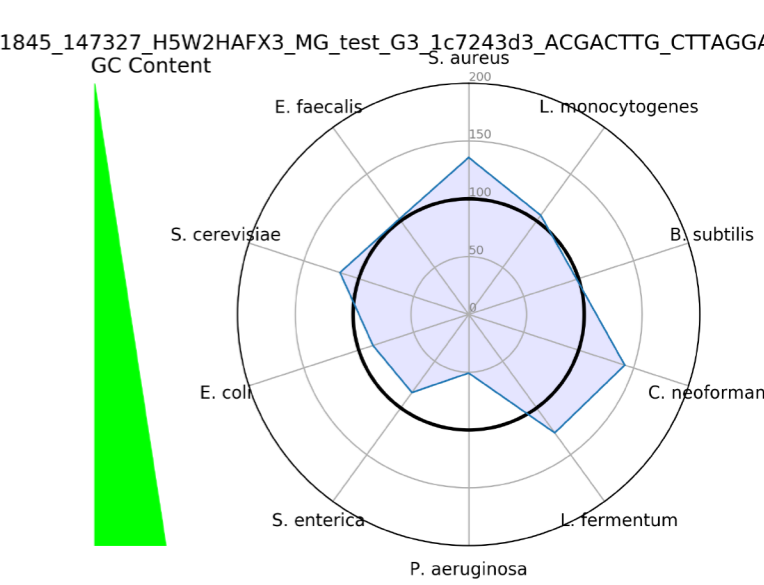

Replicate 1

Replicate 2

Replicate 3

Supplement: SupplementaryFig1_ycae075 [file supplementaryfig1_ycae075.pdf]

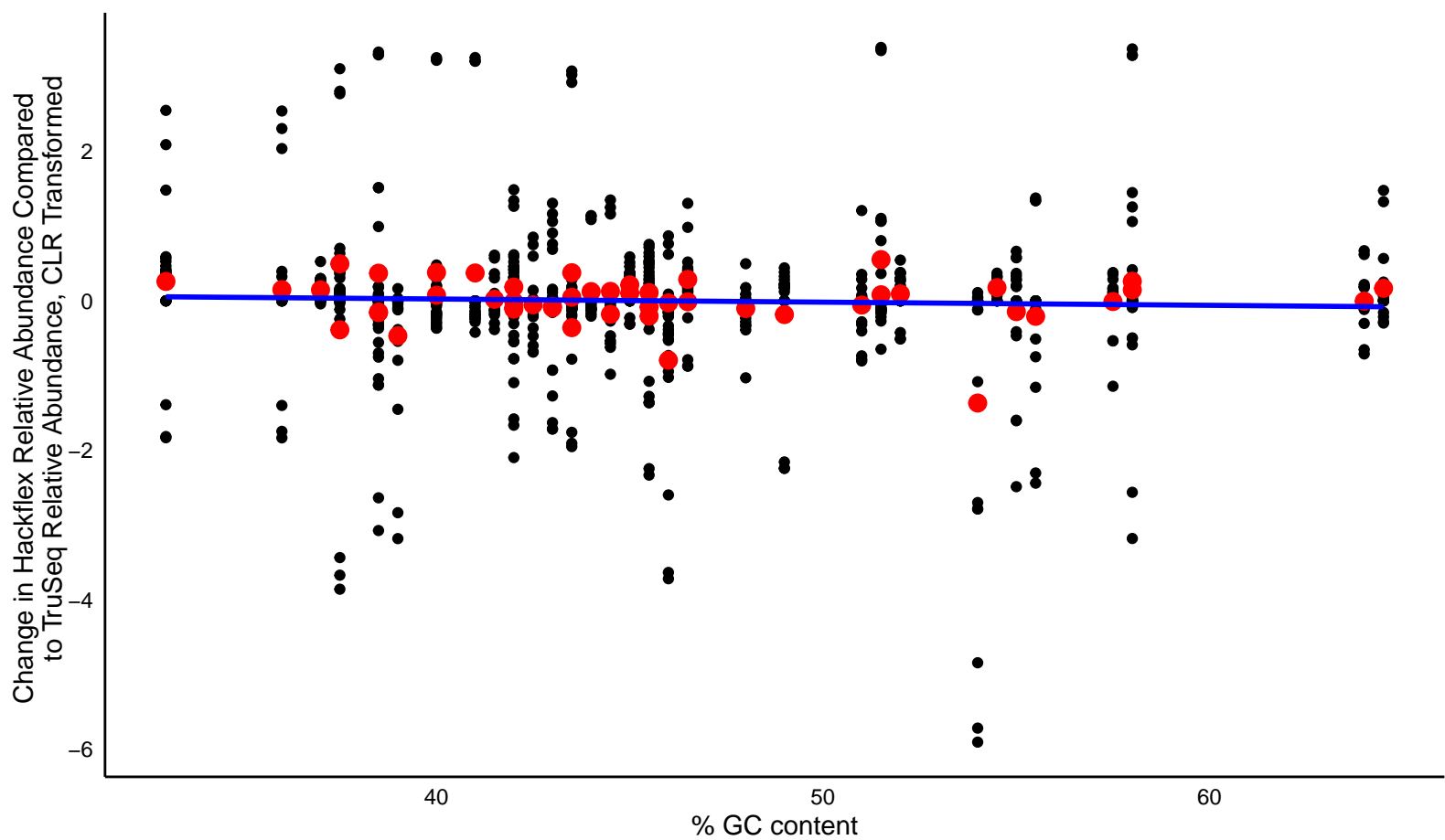

Supplement: SupplementaryFig3_ycae075 [file supplementaryfig3_ycae075.pdf]

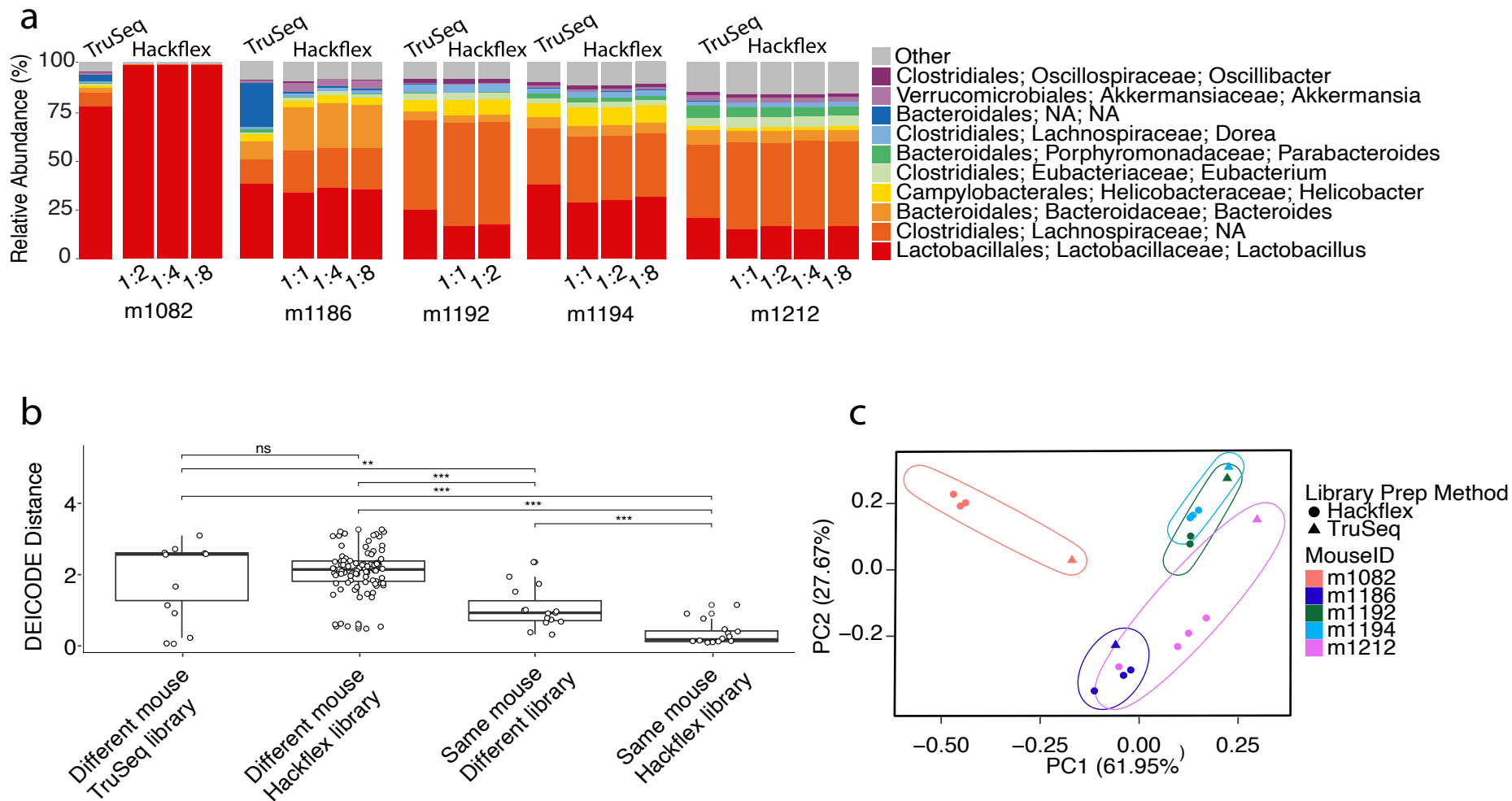

Supplement: SupplementaryFig4_ycae075 [file supplementaryfig4_ycae075.pdf]
