# Supplementary material for: Hackflex library preparation enables low-cost metagenomic profiling
Source: ISME Commun. 2024 May 29;4(1):ycae075. doi: 10.1093/ismeco/ycae075 (PMC11190725; doi:10.1093/ismeco/ycae075)
Supplement: SupplementaryFig2_ycae075 [file supplementaryfig2_ycae075.pdf]

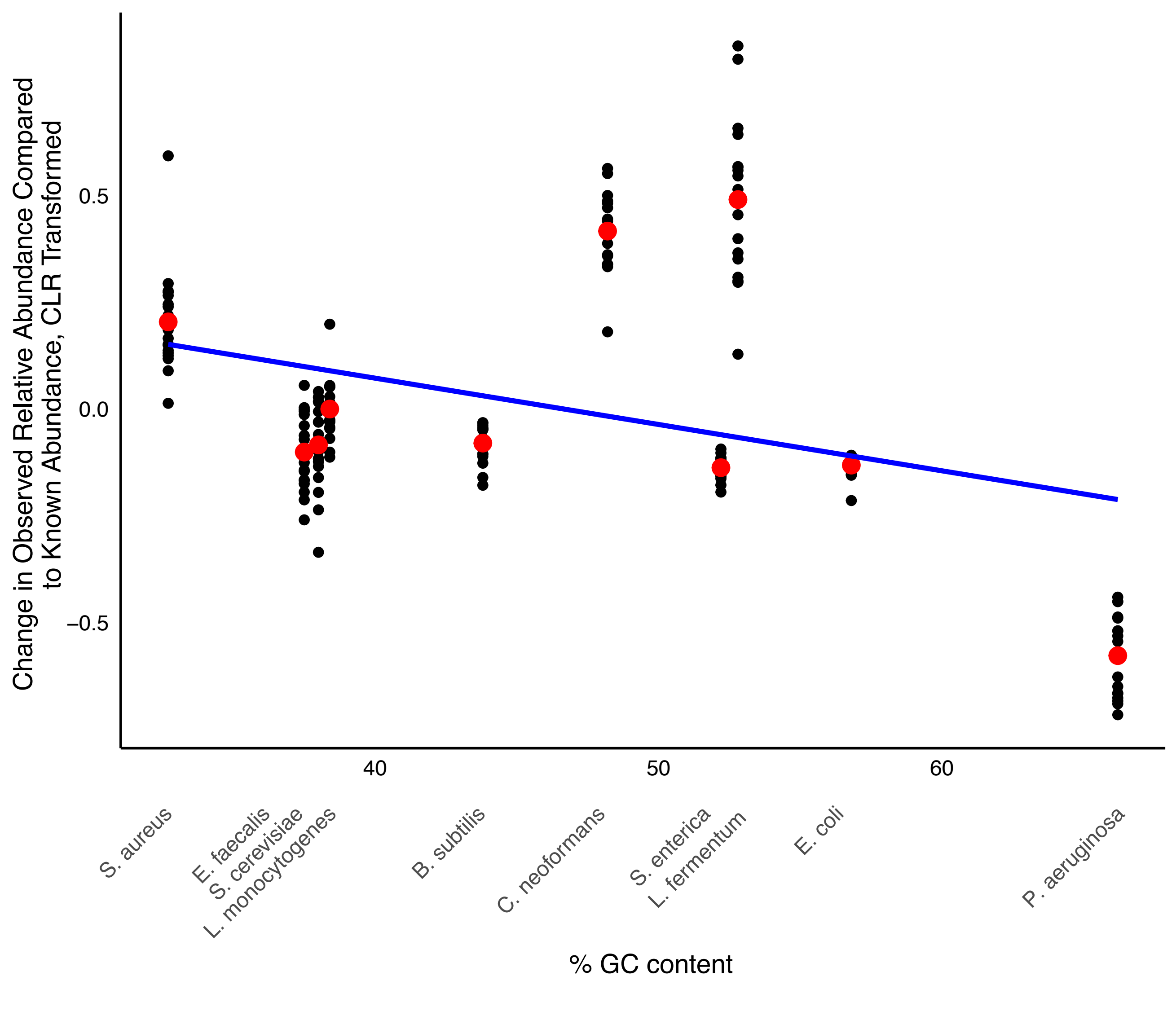

Change in Observed Relative Abundance Compared
to Known Abundance, CLR Transformed
0.5
0.0
−0.5
40
50
60
S. aureus
E. faecalis
S. cerevisiae
L. monocytogenes
B. subtilis
C. neoformans
S. enterica
L. fermentum
E. coli
P. aeruginosa
% GC content
