# Supplementary material for: Hackflex library preparation enables low-cost metagenomic profiling
Source: ISME Commun. 2024 May 29;4(1):ycae075. doi: 10.1093/ismeco/ycae075 (PMC11190725; doi:10.1093/ismeco/ycae075)
Supplement: Supplemental_Methods_ycae075 [file supplemental_methods_ycae075.docx]

**Supplemental Methods**

*Design of experiments*

To evaluate the biases of the Hackflex library preparation method, we generated and sequenced libraries from DNA from a mock microbial community. These analyses used the ZymoBIOMICS Microbial Community DNA Standard (D6306), a mixture of DNA from eight bacteria and two yeasts. The abundance of each taxon was scaled for genome size so that the DNA concentration was equal for each group. Genomic DNA from each bacterial and yeast species was at 12% relative abundance and at 2% relative abundance, respectively.

While the mock community was ideal for systematically validating Hackflex, it did not represent the complexities of a full gut microbial community. Therefore, we additionally tested fecal samples from C57BL/6 mouse (*Mus musculus domesticus*) individuals (Table S1, S4) ordered from the Jackson Laboratory (https://www.jax.org). Mice were reared under standard laboratory conditions and then a subset were transferred to an outdoor enclosure for 10 days prior to fecal sampling. All animal work was performed under Cornell University International Animal Care and Use Committee protocol number 2015-0060.

*Library preparation, pooling, sequencing, and processing*

DNA from mouse fecal samples was extracted using the Zymo Quick-DNA Fecal/Soil Microbe Miniprep Kit with bead beating. DNA was extracted from fecal samples using a magnetic bead-based protocol on an OpenTrons OT2 liquid handling robot (https://github.com/CUMoellerLab/Moeller_Opentrons_protocol_library/tree/master/Extraction/Zymo_fecal-soil_magbead). Libraries for both the mouse samples and mock DNA community were prepared using Hackflex library preparation as previously described [3]. The protocol is described below in the section “*Protocol for Hackflex library preparation*” and made available at https://github.com/CUMoellerLab/Moeller_Opentrons_protocol_library/tree/master/Library_Prep/Hackflex. TruSeq and Illumina DNA Prep libraries were prepared at the Cornell University Biotechnology Resource Center. TruSeq-Hackflex comparison libraries and Zymo mock DNA community libraries were sequenced on an Illumina NextSeq 500, and Illumina DNA Prep-Hackflex comparison libraries were sequenced on an Illumina NovaSeqX as previously described [4]. For each sequencing run (Illumina NextSeq 500 or Illumina NovaSeqX), pools of samples were multiplexed in equimolar amounts and first sequenced on an Illumina MiSeq Nano Kit v2. Data from the MiSeq Nano sequencing run was then used to infer, based on read counts, the relative abundances of each sample in the original pools. These sample DNA relative abundances were then used to re-pool DNA from each sample to equal amounts, as previously described [4], before resequencing on Illumina NextSeq 500 or Illumina NovaSeqX.

*Bioinformatic analyses of mock community samples*

Raw sequencing reads were imported into Qiita [23]. Within Qiita, adapter sequences and low-quality reads were removed using qp-fastp minimap2 (v2021.01). Processed reads were imported into QIIME 2 (v2023.9) [24]. To determine whether Hackflex recovered taxonomic groups present in the Zymo mock DNA community, we created a custom database of genomes present in the mock DNA community using Struo2 (v2.3.0) [25] and mapped reads derived from these samples using Kraken2 (v2.1.3) [15]. The number and percentage of unclassified reads for each of these samples is presented in Table S2. Taxa barplots were generated with the qiime2R package (v0.99.6) [26]. We utilized the Zymo-developed Measurement Integrity Quotient (MIQ) [17] to determine the degree to which Hackflex generated microbiota-composition measurement error within our dataset. MIQ scores were assigned by measuring the root mean square error of observed abundances compared to the known abundance of each taxon in the sample. MIQ scores can range from 0-100, with 100 indicating no bias and 0 indicating maximum bias.

Linear regression of DNA concentration and MIQ scores was performed using the lm function from base R (v4.3.2) [27]. Linear regression and ANOVA analyses to test for biases were conducted on center log ratio transformed data in base R. Correction for multiple testing (Gram status, GC content, domain) was performed using p.adjust in base R [27]. Adjusted p-values for sensitivity analyses (Table S3) were calculated separately from those calculated from the whole dataset. The summary statistics for these analyses are reported in Table S3.

*Bioinformatic analyses of mouse fecal samples*

Adapters, low-quality reads, and host-derived (mouse) sequences were removed using qp-fastp minimap2 (v2021.01) within qiita [23]. Taxonomic profiles of mouse fecal samples were estimated with Woltka (v0.1) [17], a classifier that maps reads to the Web of Life microbial phylogenetic database [24] (Tables S5, S6). Qiita-generated feature tables were imported into QIIME2 (v.2022.2) [25] and taxa bar plots were generated. DEICODE, a form of Aitchison distance that is robust to sparse datasets [20] was calculated on species-level taxonomic profiles using the qiime deicode rpca function (Fig 2B). PERMANOVA was conducted with the adonis2 function in the vegan package (v2.6.4) [29] (Table S3).

To determine whether there was a bias in relative abundance estimates driven by GC content or gram status, we tested for associations between these factors and the deviation between species’ relative abundances obtained from Hackflex and TruSeq libraries generated from the same sample. Data from TruSeq libraries was chosen for this analysis, because this library preparation strategy was expected to yield the least biased results of any library strategy tested. For any named species over 0.05103% relative abundance that was present in data generated from libraries from a given mouse prepared by both Hackflex and Truseq , we searched the name of the species on NCBI Taxonomy to obtain GC content for the reference genome for that species. We then searched species names on BacDive: The Bacteria Diversity Metadatabase (<https://bacdive.dsmz.de>) to obtain gram status. Accession numbers for the reference genomes and gram status are presented in Table S8. Relative abundance estimates were center log ratio transformed to reduce effects of compositionality, and differences in relative abundances generated by the library preparation strategies were calculated. Linear regression and ANOVA analyses to test for biases were conducted in base R. Correction for multiple testing was performed using p.adjust in base R [27]. The summary statistics for these analyses are reported in Table S3. In addition to analyses of mouse fecal microbiota based on Woltka, we also generated taxonomic profiles based on Kraken2 (v2.1.3) and Bracken (v2.9) [30]. These analyses used a Bracken kmer size of 31 and default settings (following the recommended workflow: https://github.com/jenniferlu717/Bracken).

*Protocol for Hackflex library preparation*

The protocol we used for Hackflex library preparation requires the following equipment from OpenTrons: OT-2 robot, Magnetic Module, 8-channel P300 pipette, 8-channel P10 pipette, Strip tube block (VWR half skirt PCR plate, or 3D-printed option. <https://github.com/CUMoellerLab/Moeller_Opentrons_protocol_library/tree/master/Labware/PCR_tube_block>). The following equipment is also required: thermocycler, vortex, plate centrifuge.

The following buffers and reagents are used: Magnetic-bead linked transposomes (BLT) diluted 1:40 in molecular-grade water.; 2X tagmentation buffer consisting of 20 mM Tris (pH 7.6), 20 mM MgCl, and 50% (v/v) Dimethylformamide (DMF); Tagmentation stop buffer (TSB) consisting of 0.2% sodium dodecyl sulphate (SDS); Tagemntation wash buffer (TWB) consisting of 10% polyethylene glycol (PEG) 8000 filter sterilized, 0.25 M NaCl, and Tris-EDTA buffer (TE) (10 mM Tris, 1 mM EDTA); PCR master mix consisting (per reaction) of 10 µL of 5x GXL buffer, 4 µL of 25 mM dNTPs, 2 µL of PrimeStar GXL polymerase, and 14 µL of nuclease-free water; Illumina DNA Prep–compatible index primers (a plate of unique i7 and i5); Ampure-compatible paramegnetic beads for size selection; 80% Ethanol; and molecular-grade water.

The protocol for a 96-well plate requires the following consumables: 5x VWR PCR strip tubes (89049-178), 1x NEST 15 mL reservoir plate, 1x NEST 195 mL reservoir plate, 3x BioRad 96 well 200 µL PCR plate, 2x Opentrons 300 µL tips, 1x Opentrons 200 µL filter tips, 1x Opentrons 20 µL tips, 3x Opentrons 10 µL filter tips. Note that reservoir plates can be washed and reused.

The protocol for a single plate uses the following reagents: TWB (12 mL/well), H2O (8 mL), Ampure-compatible beads (6 mL), 80% Ethanol (12 mL/well), TB1 (200 µL/tube), 1:40 diluted BLT beads (150 µL/tube), TSB (150 µL/tube), PCR Master Mix (200 µL/tube).

The protocol used is as follows:

1. Set up the robot deck in the following orientation: 96-well PCR plate in magnetic module (10), 10µL filter tips (11), 20 µL tips (7), 300 µL tips (8), 96-well PCR plate (9), 300 µL tips (4), 10 µL filter tips (5), 96-well PCR plate (6), 96-well PCR plate (1), 15 mL reservoir plate (2) strip tubes (3). Ensure DNA samples (concentration 1-100 ng/µL) are in position 1 and 300 µL tips are in position 4. Run protocol to set up the tagmentation reaction in the PCR plate on magdeck. Then remove and seal the plate. Run the TAG protocol on the thermocycler:
2. TAG protocol: Set the lid temperature to 100°C, incubate at 55 °C for 15 min, then hold at 10 °C. Centrifuge the plate, then unseal and place back on magblock. Robot will add Tagmentation Stop Buffer to stop reaction. At prompt, remove the plate, seal, and run PTC protocol (defined below) on thermocycler.
3. PTC protocol: incubate at 37 °C for 15 min, then hold at 10 °C. Centrifuge the plate, then unseal and place it back on the magblock. Robot will wash beads and add PCR master mix for library amplification. At prompt, unseal the i5 and i7 primer plates for the primer addition. Make a note of the i5 primer rotation number for the protocol. Every time the protocol is executed, it will rotate the assignment of i5 columns to samples. This will allow libraries from up to 12 sequential plates to be pooled without barcode overlap. For reference, a file recording the timestamp and i5 column rotation position is saved to the robot. At prompt, remove plate, seal, and run BLT program (defined below) on thermocycler (cycle number can be adjusted for your application if necessary).
4. BLT protocol: 3 min at 68 °C, 3 min at 98 °C, then 12 cycles of: 45 sec at 98 °C, 30 sec at 62 °C, 2 min at 68 °C, 1 min at 68 °C, then hold at 10 °C. Centrifuge the plate, unseal, and place back on the magblock.
5. Remove the sample plate from Position 1 and replace it with a new, clean BioRad PCR plate in same position. The robot will perform a large-cut size selection on the magblock, binding fragments above the desired size and transferring the small and medium fragments to the new plate. Higher PEG concentrations in the new plate will cause the medium fragments to bind to the beads.
6. At prompt, remove and discard plate from magblock, then move the plate in position 1 to the magblock. Put a new, clean PCR plate in position 1. This will be your final library plate.
7. The robot will wash and dry beads. At prompt, replace the empty tip box in position 4 with a new box of 200 µL filter tips. The robot will use these to elute DNA and transfer to the clean plate in position 1.

**Supplemental Results**

*Library fragment size and read counts*

Library pools were checked for quality on a TapeStation using ProSize Data Analysis Software. For the Hackflex-versus-Illumina DNA Prep comparisons, the mean fragment size for Hackflex libraries was 236bp and the mean fragment size for Illumina DNA Prep libraries was 501bp. For the Hackflex-versus-Illumina TruSeq comparisons, the mean fragment size for Hackflex libraries was 241bp and the mean fragment size for Illumina TruSeq libraries was 527bp. For these comparisons, >95% of library fragments prepared by Hackflex were between 100bp and 500bp, whereas >95% of library fragments prepared by Illumina DNA Prep or TruSeq were between 250bp and 1000bp, as expected. The final concentration of the pools sequenced were 1.402 ng/μL and 1.016 ng/μL for the Hackflex-versus-Illumina TruSeq comparison and Hackflex-versus-Illumina DNA Prep comparison, respectively.

In the final Zymo mock DNA community dataset, each sample yielded on average 1,207,773 reads (standard error = 59,383) before adapter and quality filtering and 926,423 reads (standard error = 40,947) after quality filtering. For the TruSeq v. Hackflex comparison experiment, each TruSeq library yielded on average 5,141,263 reads (standard error = 889,054) before adapter, host-read, and quality filtering and 3,715,651 reads (standard error = 1,001,029) after filtering. One Hackflex library sample was removed from further analyses as it yielded only one read. The remaining Hackflex-prepared samples yielded an average of 1,314,593 reads (standard error = 51,575) per sample before adapter and quality filtering and 638,986 (standard error = 75,094) reads per sample after filtering. For the Illumina DNA Prep versus Hackflex comparison experiment, the Illumina DNA Prep libraries yielded on average 8,575,658 reads (standard error = 2,100,353) per sample before adapter, host-read, and quality filtering and 7,652,590 reads (standard error = 1,897,295) after filtering. The Hackflex libraries yielded on average 4,143,779 reads (standard error = 276,978) per sample before adapter, host-read, and quality filtering and 2,311,362 reads (standard error = 236,831) after filtering. The lower coverage for Hackflex libraries relative to TruSeq or Illumina DNA Prep libraries enabled additional technical replication for Hackflex libraries. In both comparisons, <50,000 reads were observed for the negative control samples (i.e., blank wells containing no template DNA but for which library preparation was conducted), consistent with minimal contamination in our library preparations.

*Hackflex was not biased as a function of microbial domain, gram status, GC content, or genome size*

To determine whether results from Hackflex libraries are biased as a function of domain (Bacteria vs Eukaryota), gram status (for bacterial taxa), GC content, or genome size, we performed ANOVAs (for domain and gram status) and linear regressions (for GC content and genome size) based on the deviation between centered log ratio transformed relative abundances of each Zymo species with each of these traits. These analyses revealed no significant effects of domain, gram status, GC content, or genome size after accounting for multiple testing (Table S3, Figure S2, Figure S3). A modest effect of genome size was observed when only bacteria were considered, but sensitivity analyses showed that the significance of this result (at the p-value <0.05 level) was dependent on a single species (*L. fermentum*). Together, these results fail to identify any biases of data obtained by Hackflex library preparation method as a function of domain, gram status, GC content, or genome size.

*Hackflex corroborates TruSeq when applied to mouse gut metagenomes*

We also tested the accuracy of Hackflex for biological samples (mouse fecal metagenomes) by comparing its performance with that of TruSeq, a widely used and more costly method for metagenomic sequencing. We prepared 15 total libraries from five fecal samples from five different mice, with between two and four 1:2 dilutions per Hackflex-prepared sample (Fig S4A, Table S1). We used a homemade TruSeq equivalent method that uses the same ligase as the commercial Illumina TruSeq library prep [31]. For downstream analyses, TruSeq samples were used as references against which Hackflex samples were compared for accuracy. Given the higher library costs, we sequenced TruSeq libraries at approximately 4x the depth of Hackflex and prioritized technical replication for Hackflex libraries.

Hackflex performed comparably to TruSeq in recovering metagenomes from mouse fecal samples (Figure S4A). PERMANOVA based on Woltka [19] Web of Life [23] taxonomic profiles generated for all samples indicated that mouse ID was a stronger driver of community variation (R^2^ = 0.309) compared to library prep method (TruSeq vs. Hackflex) (R^2^ = 0.151) (Table S2). However, in one mouse fecal sample dominated by *Lactobacillus* (m1082), Hackflex failed to recover the remaining diversity in this sample. This result is consistent with the results from Zymo mock DNA communities that indicated a bias towards *Lactobacillus*. Pairwise DEICODE comparisons between samples showed that taxonomic profiles of samples collected from the same mouse but whose libraries were prepared by different methods were more compositionally similar than were those of samples collected from different mice but whose libraries were prepared by the same method (Figure S4) (p < 0.001, permutation t-test for non-independent samples, Bonferroni multiple comparison correction). Furthermore, principal coordinate analysis of robust Aitchison distances using DEICODE [20] showed that these samples clustered by mouse individual rather than library preparation method (Figure S4C). Interestingly, the two mice that were the least distinguishable, m1192 and m1194, were cagemates and therefore expected to share many microbes [21] (Table S4). We did not observe any bias with regard to GC content (Bonferroni-corrected *p-*value = 0.197) (Figure S3) or gram status (Bonferroni-corrected *p*-value = 1) (Table S4). Similar results were observed based on analyses of Kraken/Bracken classified reads (Table S7).

**Supplementary References**

23. Gonzalez A, Navas-Molina JA, Kosciolek T, McDonald D, Vázquez-Baeza Y, Ackermann G, et al. Qiita: Rapid, web-enabled microbiome meta-analysis. Nature Methods. 2018; 15(10):796–8. doi:10.1038/s41592-018-0141-9

24. Zhu Q, Mai U, Pfeiffer W, Janssen S, Asnicar F, Sangers JG, et al. Phylogenomics of 10,575 genomes reveals evolutionary proximity between domains Bacteria and Archaea. *Nat Commun*. 2019; 10(1):5477. Published 2019 Dec 2. doi:10.1038/s41467-019-13443-4

25. Bolyen E, Rideout JR, Dillon MR, Bokulich NA, Abnet CC, Al-Ghalith GA, et al. Reproducible, interactive, scalable and extensible microbiome data science using QIIME 2. *Nature Biotechnology*. 2019; 37(8):852–7.

26. Youngblut ND, Ley RE. Struo2: efficient metagenome profiling database construction for ever-expanding microbial genome datasets. *PeerJ*. 2021;16;9:e12198. doi: 10.7717/peerj.12198.

27. Bisanz JE. qiime2R: Importing QIIME2 artifacts and associated data into R sessions. 2018. <https://github.com/jbisanz/qiime2R>.

28. R Core Team. R: A Language and Environment for Statistical Computing. 2023. R Foundation for Statistical Computing, Vienna, Austria. <https://www.R-project.org/>.

29. Oksanen J, Simpson G, Guillaume Blanchet F, Kindt R, Legendre P, Minchin PR, *et al.* vegan: Community Ecology Package. R package version 2.6-4. 2022. https://CRAN.R-project.org/package=vegan.

30. Lu J, Breitwieser FP, Thielen P, Salzberg SL. Bracken: estimating species abundance in metagenomics data. *PeerJ Computer Science*. 2017; 3:e104.

31. Rohland N, Reich D. Cost-effective, high-throughput DNA sequencing libraries for multiplexed target capture. *Genome Research*. 2012; 22. doi:10.1101/gr128124.111

**Supplementary Figure and Table Captions**

**Figure S1.** MIQ score diagrams for each mock DNA community sample. Rows group samples by the concentration of DNA. Black circles in each diagram represent expected relative abundances . Values outside or inside the black circle indicate overrepresentation and underrepresentation, respectively. Microbes are arranged around each circle by GC content.

**Figure S2**. Hackflex shows no bias with regard to GC content. Linear regression of mock DNA community samples sorted by GC content shows that Hackflex has no bias towards lower GC content (slope = -0.010856, t-statistic = -1.125, Bonferroni-corrected p-value p = 1). Red points are average difference for a given species.

**Figure S3.** In a complex community, Hackflex shows no bias with regard to GC content in comparison to TruSeq. Linear regression of named species in mouse samples sorted by GC content shows that Hackflex is unbiased with regards to GC content in this community (slope = 0.008952, t-statistic = 0.643, Bonferroni-corrected p-value = 0.197). Red points are average difference for a given species.

**Figure S4.** Hackflex recovers individual signatures in mouse gut metagenomes. A) Stacked barplots show relative abundances of microbial taxa observed in mouse metagenomes sequenced from libraries prepared with TruSeq or Hackflex. Colors denote microbial genera as indicated by the key. B) Boxplots show the DEICODE Aitchison dissimilarities between pairs of samples from different mice prepared with TruSeq, different mice prepared with Hackflex, the same mice prepared with different library methods, and the same mouse prepared with Hackflex. Points represent pairwise comparisons between samples. Asterisks and ‘ns’ indicate significance of differences between boxplots based on permutation t-tests for non-independent samples; Bonferroni-corrected *p*-value < 0.01 **; < 0.001 ***; > 0.05 ns. C) Principal coordinate analysis plots show Robust Aitchison dissimilarities among samples. Circles represent Hackflex-prepared libraries and triangles represent TruSeq-prepared libraries. Colors denote individual mice as indicated by the key.

**Table S1. Sample metadata.**

**Table S2. Mock DNA Community MIQ Scores.**

**Table S3. PERMANOVA formulae and tables.**

**Table S4. Mouse metadata.**

**Table S5. Woltka taxonomy table for Illumina DNA Prep and Hackflex comparisons.**

**Table S6. Woltka taxonomy table for TruSeq and Hackflex comparisons.**

**Table S7. Kraken2/Bracken taxonomy table for all samples.**

**Table S8. Reference genomes used for bias analyses.**
